# Case of Yellow Fever Occurring in St. Louis, August, 1855

**Published:** 1856-01

**Authors:** Wm. Webb


*Case of Yellow Fever occurring in St. Louis, August*, 1855. By Wm. Webb, M. D.

The subject of the contagion and importation of Yellow Fever, still being debatable with the profession, it behooves us to put upon record, all such well attested facts, as may be of service in rendering a solution of this much discussed and important question.

To fully understand any disease, it must be viewed in every possible aspect. We must study its cause, history, nature, treatment, its modifications by individuals, age, sex, temperament, season, climate, country, endemic, epidemic and sporadic. With this object in view, I propose to give a sketch of a case of yellow fever, that has fallen under my observation, hoping that even this mite collected into the treasury of science may not be without some interest in elucidating this abstruce subject. I was called about six o'clock in the afternoon of Sunday, August 14th, to see Mrs. S., an Irish woman, aged about twenty-two years, advanced to the eighth month of pregnancy with her second child; fair skin, light hair, blue eyes, well made, and remarkably healthy—not having been at all sick during a residence of five years in St. Louis, with the exception of a slight attack of intermittent fever some three years since. She has not been absent from her residence on Sixth street, between Franklin avenue and Wash, for many weeks, except to market, a few squares distant—was taken sick the day previous to my visit, just after her return from market, with severe pain in the head, back, and limbs, and fever. Found her about thirty hours after her attack, with considerable fever; skin hot; tongue coated with a whitish fur; pulse 120; respiration hurried; other symptoms not noted as I diagnosed remittent fever. Ordered quinine, fifteen grains; blue mass, ten grains; pulv. opium, two grains.

Monday, the third day of attack, found her up walking across the floor, says that she feels much better; rested well during the night; pulse and skin considered quite natural; did not note other symptoms particularly, though her respiration seemed to be greatly embarrassed, which was attributed to her situation. Ordered ten grains of the sulphate of quinine, and directed her to keep quiet and in bed. Monday evening—did not see her, but prescribed an oleaginous mixture, as her bowels had not been moved. Was summoned about five o'clock, Tuesday morning to see her, had been very comfortable until about three o'clock in the morning, when she was taken with vomiting; had taken some tea and thought at first that it was only the tea vomited, soon afterwards detected stains on the sheets that looked bloody; says, that "she only spits it up." Found her stupid, though she was perfectly conscious when aroused, and seemed anxious about her condition; no urine for several hours; no stool; pulse 100.—The matter emitted looked like an infusion of senna. Ordered an injection of soap and water. Ten o'clock—has had several evacuations from the bowels; did not see them, but learned from the nurse that they were consistent and of a light color; has passed some urine; still stupid, restless, and alarmed about her situation; has been belching up a fluid, every few minutes, in quantities from a tablespoon to a wine-glassful, very much like a strong infusion of coffee, with shreds and flocculi in it; getting darker and darker, every hour; tongue very red, dry and shining underneath; pulse 100, weaker; skin around the neck and chest considered unnaturally yellow; eyes very much injected and red, tunica albuginea was of a *bright yellow*, and in striking contrast to the injected blood vessels. Now diagnosed yellow fever—12 o'clock, received a vial containing some of the matter then emitted, darker and thicker than before. Two o'clock, met Dr. McPheeters in consultation. She was now very stupid, almost comatose; all previous symptoms aggravated; tongue stained as black as charcoal on the upper part, the rest very red, dry and shining; vomit black as tar, and looked exactly like coffee grounds, was spit up by the cupful, once it was ejected several feet in a stream; yellowness was remarked upon the neck, chest, and tunica adnata, by Dr. McP.; no urine; no labor pains. Diagnosis, yellow fever—prognosis—death inevitable. Gave some general directions, and ordered ten drops every half hour of the tincture of the muriate of iron. Never vomited again. At three o'clock, labor commenced and in a hour she was delivered of a stillborn child, small, but well developed; skin natural; no hemorrhage; abdomen flabby; uterus did not contract well. At four o'clock, she was in collapse; skin cold and clammy, pulseless, unconscious, breathed hurriedly and with great difficulty; expired at a quarter to five o'clock. No autopsy.

I handed Professor Cooper, two days afterward, for microscopic examination, some of the matter ejected five hours before death, and he has handed me the following note:

"The liquid is in a state of putrefaction, notwithstanding, the vegetable parasite the *sarcina ventriculi* of Goodsir—the *merismopœdia ventriculi* of Ch. Robin—*are seen in great quantity.* This vegetable parasite is not affected by putrescence."

*Remarks.*—Although the symptoms were not noted as minutely as we would have wished, still enough has been recorded to stamp this at once a case of yellow fever. We merely state the facts as they were remarked by us and leave them to interpret themselves.— *St. Louis Med. & Surg. Journal.*

